# Barriers and Facilitators to AI Implementation in Intensive Care Units in China: Qualitative Study Among Nurse Managers

**DOI:** 10.2196/96172

**Published:** 2026-08-18

**Authors:** Wenshuo Dong, Yu Xia, Lichao Kan, Lei Wei, Xiaofei Kang, Yan Dong, Min Ding

**Affiliations:** 1 Department of Critical Care Medicine, Shandong Provincial Hospital Affiliated to Shandong First Medical University, No. 324, Jingwu Weiqi Road, Huaiyin District, Jinan City, Jinan, Shandong, China, +86 15168863616; 2 School of Nursing, Shandong Xiandai University, Jinan, China; 3 Department of Geriatric Medicine (Health Care Department), Shandong Provincial Hospital Affiliated to Shandong First Medical University, Jinan, Shandong, China

**Keywords:** AI, barriers, facilitators, Consolidated Framework for Implementation Research, CFIR, qualitative

## Abstract

**Background:**

AI has shown significant potential in intensive care unit (ICU) nursing practice, enhancing efficiency, decision-making, and patient safety. However, evidence regarding the implementation factors of AI in ICU nursing remains limited, particularly from the perspective of nursing leadership.

**Objective:**

This study aimed to explore the perceived barriers and facilitators to the implementation of AI in ICUs in China from the perspectives of ICU nurse managers, guided by the Consolidated Framework for Implementation Research (CFIR).

**Methods:**

A qualitative study using semistructured, face-to-face interviews was conducted with 11 ICU nurse managers from tertiary hospitals across 7 geographic regions in China from August to October 2025. Participants were recruited through maximum variation purposive sampling and approached via WeChat (Tencent) or telephone. Interview questions were informed by the CFIR framework. Data collection and analysis were conducted iteratively until data saturation was reached. Data were audio-recorded, transcribed verbatim, and analyzed using directed content analysis guided by the CFIR.

**Results:**

A total of 20 factors were identified across 5 CFIR domains, including 5 barriers, 13 facilitators, and 2 neutral influencing factors. Key barriers included high implementation costs, limited adaptability and complexity of AI systems, ethical and privacy concerns, shortages of interdisciplinary talent, and communication challenges between clinical and technical teams. Major facilitators encompassed perceived relative advantages of AI, supportive national policies, leadership engagement, a positive implementation climate, readiness for implementation, and nurses’ self-efficacy.

**Conclusions:**

Addressing the complexity of AI systems, their limited fit with clinical contexts, and the shortage of interdisciplinary expertise is critical for successful implementation. Hospital administrators and health policymakers should optimize resource allocation, strengthen AI-related training for health care professionals, and develop context-specific implementation strategies to promote the effective, appropriate, and sustainable use of AI in critical care nursing practice.

## Introduction

In intensive care units (ICUs), nurses play a central role in continuous patient monitoring, early detection of deterioration, and coordination of multidisciplinary care. However, ICUs globally face persistent challenges such as nursing workforce shortages, increasing care complexity, and rising demands for high-quality and efficient care [1]. Within this context, AI has been widely applied in ICUs [2]. AI technologies, including early warning systems [3], clinical decision support tools [4], prediction models [5], and AI robots [6], have demonstrated significant potential in enhancing patient safety, reducing adverse events, optimizing workflows, and supporting clinical decisions in critical care settings [7-9].

Despite these potential advantages, the implementation of AI in ICU settings still faces considerable challenges. A systematic review noted that although there has been a significant increase in AI research in critical care medicine in recent years, application from development to clinical implementation remains limited [10]. The translation of AI technologies into routine clinical practice is constrained by several significant barriers, including difficulties in integrating AI into existing workflows, the heterogeneity and fragmentation of clinical data, and unresolved ethical and regulatory issues [11]. Importantly, this process is not purely technical. Successful implementation is shaped by multiple interacting factors at the intervention, organizational, individual, and process levels. In complex and high-risk environments such as ICUs, neglecting these multilevel determinants may hinder adoption, limit sustained use, and reduce the anticipated benefits of AI systems.

Nurses are the primary users and key stakeholders in AI implementation [12,13]. Notably, ICU nurse managers play a key role in decision-making, resource allocation, workflow redesign, and staff training related to new technologies [14]. Their perspectives are critical for understanding implementation challenges and opportunities. Previous studies have explored the facilitators and barriers to the implementation of AI among nurses in general health care settings [13,15,16]. These studies focused on factors such as perceived benefits, organizational support, technical limitations, ethical concerns, and digital literacy. However, the existing evidence has predominantly focused on frontline nurses or nursing students, has limited representation of ICU environments, and has given limited consideration to nurse managers’ roles in technology implementation. Given the unique characteristics of ICUs, including high patient acuity, complex workflows, and time-sensitive clinical decision-making, implementation determinants identified in other health care settings may not be fully generalizable to the ICU context. This gap limits a comprehensive understanding of how leadership perspectives and critical care contexts shape AI integration in nursing practice.

The Consolidated Framework for Implementation Research (CFIR) offers a robust and systematic approach to examining multilevel factors that influence the implementation of complex interventions in health care settings [17]. The CFIR is composed of 5 major domains: intervention characteristics, outer setting, inner setting, characteristics of the individuals involved, and the process of implementation [18]. CFIR has been widely applied to explore barriers and facilitators of health technology implementation in complex health care settings [19-22]. Therefore, this study aimed to explore the perceived barriers and facilitators to the implementation of AI in ICU in China from the perspectives of ICU nurse managers, using the CFIR as a guiding framework. By identifying key determinants across multiple CFIR domains, this study seeks to provide evidence-informed insights to support effective and context-sensitive AI implementation in Chinese ICUs and similar health care settings.

## Methods

### Study Design and Theoretical Framework

This study used a qualitative design using semistructured interviews to explore barriers and facilitators to the implementation of AI in ICUs in China from the perspectives of ICU nurse managers. The CFIR was used as the guiding theoretical framework to inform the overall study design. Specifically, CFIR domains and constructs guided the development of the interview guide to ensure comprehensive coverage of multilevel implementation determinants and further provided an analytic lens for organizing and interpreting findings. The study focused on identifying perceived facilitators and barriers across CFIR domains, thereby generating a structured understanding of determinants influencing AI implementation in Chinese ICU settings. This study’s reporting followed the rules for COREQ (Consolidated Criteria for Reporting Qualitative Research; Checklist 1 [23]).

### Setting and Sample

This study was conducted in ICUs of tertiary hospitals in China. To capture diverse implementation contexts across China, we adopted a maximum variation purposive sampling strategy [24]. According to the standard regional divisions commonly used in China, the country was stratified into 7 regions: Northeast, North China, East China, Central China, South China, Southwest, and Northwest. Within each region, ICU nurse managers from tertiary hospitals were invited to participate in order to capture diverse perspectives on the implementation of AI in ICU practice, including perceived barriers and facilitators across different organizational contexts.

Eligibility requirements for participants were as follows: (1) currently serving as ICU nurse managers with responsibilities for unit-level nursing administration and oversight of ICU workflows and management practices; (2) at least 5 years of ICU management experience; and (3) involvement in or knowledge of the implementation of AI or information systems within their units. Based on these criteria, the research team intentionally selected eligible nurse managers from tertiary hospitals in 7 geographic regions of China as interview participants. Potential participants were approached via WeChat (Tencent) or by phone. During the initial contact, researchers briefly explained the study’s objectives, procedures, and the voluntary nature of participation. Those who agreed to participate were subsequently scheduled for a face-to-face interview at a time and location convenient for them. Before each interview, the researcher provided additional study information, answered participants’ questions, and obtained written informed consent. Participants were assigned unique identifiers to protect confidentiality.

The sample size was determined through an iterative assessment of data saturation during data collection and preliminary analysis [25]. Data collection and coding were conducted concurrently. After each round of interviews, 2 researchers (WD and YX) independently reviewed the transcripts, preliminary codes, and CFIR construct mapping to determine whether new information continued to emerge. The research team maintained a saturation tracking grid throughout the interviews. After the ninth interview, only minor refinements to existing codes were identified, and no new CFIR constructs emerged. The tenth and eleventh interviews confirmed previously identified barriers and facilitators without generating substantially new codes, items, or constructs. Recruitment ceased after 11 interviews when the research team agreed that data saturation had been achieved.

### Data Collection

A semistructured interview guide was created, with all questions revolving around the CFIR. The questions focused on current practices of AI in ICUs, attitudes toward AI, and its barriers and facilitators (Multimedia Appendix 1). This interview guide was reviewed by qualitative research experts and pretested with 3 ICU head nurses, followed by revisions to ensure clarity and relevance. Data were collected between August and October 2025. Interviews were conducted by trained researchers (WD and YX) with experience in qualitative methods and clinical nursing research. All interviews were conducted face-to-face in a quiet and private room. No prior relationship existed between the interviewers and participants before data collection. With participants’ consent, all interviews were audio-recorded to ensure accurate data capture. All recordings were transcribed verbatim in Chinese shortly after the interviews. Data analysis was performed using the original Chinese transcripts. Selected excerpts were translated into English after data analysis by a bilingual researcher and independently checked by a second bilingual researcher. Discrepancies were resolved through discussion to ensure accuracy and conceptual equivalence. Selected excerpts were back-translated to verify fidelity of meaning. To address potential bias, interviewers engaged in reflexive practices, including maintaining reflective journals.

### Data Analysis

The data were analyzed using directed content analysis [26,27], combining data-driven inductive coding with theory-driven deductive interpretation guided by the CFIR framework. Researchers repeatedly reviewed transcripts to familiarize themselves with the data, extracting meaningful text units, such as sentences, paragraphs, and words for coding through inductive methods. Similar codes were subsequently grouped into items. These inductively derived items were deductively mapped onto the domains and constructs of the CFIR framework. For example, a participant statement, such as “Third, it reduces labor costs by freeing nurses from repetitive, mechanical tasks,” was initially coded and grouped as the “Mitigating labor shortages” item and subsequently mapped to the CFIR construct relative advantage. Following the coding and mapping of data to CFIR constructs, the researchers assigned ratings to each construct, indicating whether it was a barrier, a facilitator, or a neutral factor [18,28]. Construct ratings were determined according to both valence and strength [18,29]. Valence indicated whether a construct predominantly facilitated or hindered AI implementation, whereas strength reflected the degree of its influence. When both positive and negative views were identified within the same CFIR construct, all relevant coded segments were retained and reviewed. The overall valence of the construct was determined first, followed by its strength. The researchers considered the level of agreement among participants, the strength and consistency of the language used, the presence of concrete examples, and the stated influence of the construct on implementation or related constructs. If positive and negative influences were approximately balanced, or if divergent perspectives did not indicate a predominant direction, the construct was rated as neutral (0). If one direction predominated but the supporting evidence was limited, mixed, or general, the construct was rated +1 or –1. If one direction clearly predominated and was supported by consistent, specific, and concrete evidence, the construct was rated +2 or –2. The rating criteria used in this study are presented in Table 1. Information extraction, transcript coding, and construct rating were all carried out independently by 2 researchers (WD and YX). In cases of disagreement, a third researcher (LW) was consulted to reach consensus. Interrater agreement for construct ratings was assessed using weighted κ, which was 0.87. The final results were reviewed and confirmed at a meeting of the research team. NVivo software (Lumivero) was used to support qualitative data analysis by organizing interview transcripts, managing coding structures, grouping codes within CFIR domains and constructs, and facilitating the retrieval and comparison of coded segments across participants. The software served as an analytical support tool, while all coding, interpretation, and rating decisions were made by the research team.

**Table 1.** Criteria used to assign ratings to constructs.

Rating	Criteria
−2	The construct is a negative influence in the organization, an impeding influence in work processes, and/or an impeding influence in implementation efforts. The majority of interviewees (at least 2) describe explicit examples of how the key or all aspects (or the absence) of a construct manifests itself in a negative way.
−1	The construct is a negative influence in the organization, an impeding influence in work processes, and/or an impeding influence in implementation efforts. Interviewees make general statements about the construct manifesting in a negative way but without concrete examples: The construct is mentioned only in passing or at a high level without examples or evidence of actual, concrete descriptions of how that construct manifests. There is a mixed effect of different aspects of the construct but with a general overall negative effect. There is sufficient information to make an indirect inference about the generally negative influence. Judged as weakly negative by the absence of the construct.
0	A construct has neutral influence if: It appears to have neutral effect (purely descriptive) or is only mentioned generically without valence. There is no evidence of positive or negative influence. Credible or reliable interviewees contradict each other. There are positive and negative influences at different levels in the organization that balance each other out. Different aspects of the construct have a positive influence while others have a negative influence, and overall, the effect is neutral.
+1	The construct is a positive influence in the organization, a facilitating influence in work processes, and/or a facilitating influence in implementation efforts. Interviewees make general statements about the construct manifesting in a positive way but without concrete examples: The construct is mentioned only in passing or at a high level without examples or evidence of actual, concrete descriptions of how that construct manifests. There is a mixed effect of different aspects of the construct but with a general overall positive effect. There is sufficient information to make an indirect inference about the generally positive influence.
+2	The construct is a positive influence in the organization, a facilitating influence in work processes, and/or a facilitating influence in implementation efforts. The majority of interviewees (at least 2) describe explicit examples of how the key or all aspects of a construct manifest themselves in a positive way. Missing Interviewees were not asked about the presence or influence of the construct; or if asked about a construct, their responses did not correspond to the intended construct and were instead coded to another construct. Interviewees’ lack of knowledge about a construct does not necessarily indicate missing data and may instead indicate the absence of the construct.

### Ethical Considerations

This study was conducted in accordance with the principles of the Declaration of Helsinki. Ethical approval was obtained from the Ethics Committee of Shandong Provincial Hospital (SWYX: number 2025‐888). All participants were informed of the study purposes and signed consent forms. Individual interviews were audiotaped and transcribed if participants agreed. Identifiable information was removed during transcription to protect participants’ anonymity. All research data were stored on password-protected laptops. To prevent disclosure of respondents’ identities, the transcribed data exclude potentially identifying information such as sex, age, professional title, and specific job roles.

## Results

### Characteristics of Participants

A total of 13 eligible ICU nurse managers were invited to participate in this study; 11 completed face-to-face interviews and 2 did not participate: one due to a scheduling conflict and the other due to illness. The interviews ranged from 27 to 78 minutes. The average age of participants was 45.4 (SD 4.5) years (ranging from 34 to 51 y). Half of the participants held a master’s degree, and 90.9% (10/11) were female participants. Overall, 90.9% (10/11) of participants had more than 15 years of management experience and 36.4% (4/11) were associate chief nurses. Participants N1-N3 reported experience with or exposure to several AI-related technologies in their ICUs, including early risk warning systems, consumables management systems, logistics robots, large language models, wearable devices, virtual simulation training systems, intelligent access control systems, intelligent call systems, and others. Detailed demographic characteristics of the participants are presented in Table 2.

**Table 2.** Demographic characteristics of the participants.

ID	Sex	Age (y)	Education level	Job title	Working experience (y)	Management experience (y)	Geographical area
N1	Female	34	Master	Associate chief nurse	8	5	Southwest China
N2	Female	45	Master	Chief nurse	23	15	Southwest China
N3	Female	45	Master	Chief nurse	24	15	Northwest China
N4	Female	51	Bachelor	Chief nurse	33	26	Central China
N5	Female	46	Master	Associate chief nurse	26	19	Northeast China
N6	Female	45	Bachelor	Associate chief nurse	27	15	East China
N7	Male	44	Bachelor	Associate chief nurse	22	17	East China
N8	Female	46	Bachelor	Chief nurse	26	16	East China
N9	Female	47	Bachelor	Chief nurse	24	17	South China
N10	Female	47	Master	Chief nurse	27	16	East China
N11	Female	51	Master	Chief nurse	33	21	North China

### Summary of the Barriers and Facilitators

**Figure 1.**
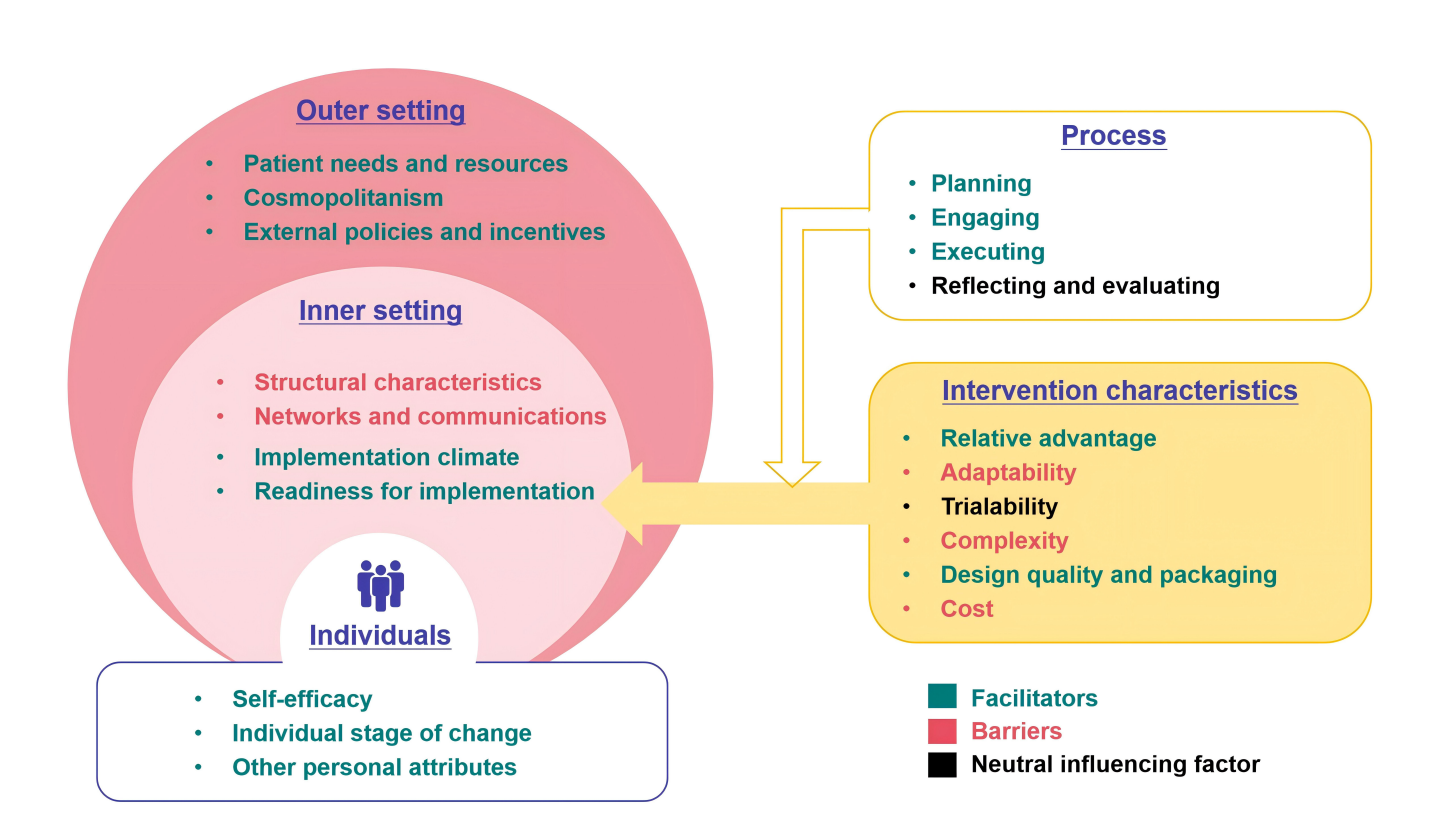
Barriers and facilitators based on the Consolidated Framework for Implementation Research (CFIR) framework.

This study identified 36 items mapped across the 5 domains and 20 constructs of the CFIR framework. Overall, these constructs were categorized into 5 barriers, 13 facilitators, and 2 neutral influencing factors. Figure 1 and Table 3 summarize these findings. Detailed information can be found in Multimedia Appendix 2. The following sections present the results in detail, with particular attention to factors specific to the Chinese context.

**Table 3.** Barriers and facilitators to the implementation of AI in intensive care units^a^.

CFIR^b^ domain and CFIR constructs	Items	Ratings
Intervention characteristics		
Relative advantage (+)	Enhancing nursing efficiency and convenience Mitigating labor shortages Providing information integration and decision support Enhancing nursing quality and patient safety Optimizing teaching and training	+2
Adaptability (–)	System optimization and algorithm iteration Scenario adaptation and adjustment	–1
Trialability (±)	—^c^	0
Complexity (–)	Insufficient system algorithm and clinical adaptability Judgment conflicts and dependency risks Humanistic concern and ethical challenges Data security and privacy protection System integration and data organization challenges	–2
Design quality and packaging (+)	—	+2
Cost (–)	—	–2
Outer setting		
Patient needs and resources (+)	—	+1
Cosmopolitanism (+)	—	+2
External policies and incentives (+)	—	+2
Inner setting		
Structural characteristics (–)	Shortage of multidisciplinary nursing information professionals IT team development	–1
Networks and communications (–)	Interdisciplinary collaboration Informal communication Interdisciplinary communication barriers	–1
Implementation climate (+)	Incentive bonuses and system orientation Role model influence and team learning	+2
Readiness for implementation (+)	AI knowledge training Leadership engagement	+1
Characteristics of individuals		
Self-efficacy (+)	—	+1
Individual stage of change (+)	—	+2
Other personal attributes (+)	—	+2
Process		
Planning (+)	Clinical nursing practice Nursing environment and humanistic optimization Education, training, and scientific research innovation	+2
Engaging (+)	—	+2
Executing (+)	—	+2
Reflecting and evaluating (±)	—	0

a “+” refers to a facilitator, “–” refers to a barrier, and “±” refers to a neutral influencing factor. CFIR construct ratings indicate the direction and strength of influence on AI implementation: +2=strong facilitator (the majority provides concrete positive examples), +1=weak facilitator (a general positive influence, with few examples), 0=neutral influence, –1=weak barrier (a general negative influence, with few examples), –2=strong barrier (the majority provide concrete negative examples).

b CFIR: Consolidated Framework for Implementation Research.

c Not applicable.

### Intervention Characteristics

In this study, intervention characteristics referred to nurses’ perceptions and experiences regarding the intrinsic attributes of AI systems. Six constructs were identified as influencing the implementation of AI in ICUs, including 3 barriers, 2 facilitators, and 1 neutral influencing factor (Table 3).

#### Barriers: Adaptability, Complexity, and Cost

Participants reported that AI applications required continuous system optimization and algorithmic iteration to function effectively, particularly during the early stages of implementation. False alarms were perceived as common initially, although participants expected their frequency to decrease as the volume of training data increased. Additionally, difficulties related to contextual and scenario-specific adaptation were frequently mentioned, limiting the applicability of AI systems across different ICUs.

*[False alarms] They will occur initially, but as the volume of data increases, the frequency of false alarms will decrease.*

[N1]

*Software that works well at one hospital may not necessarily be suitable for your own hospital.*

[N8]

Beyond adaptability, many participants emphasized the inherent complexity and potential risks associated with AI use in clinical practice. Current AI algorithms were perceived as insufficiently capable of managing complex or atypical clinical situations, which sometimes resulted in false-positive or false-negative alerts. Ethical and humanistic concerns were also raised, with participants noting that AI systems were inherently impersonal and could not replace human-centered nursing care. Additionally, concerns regarding data security and patient privacy further contributed to the perceived complexity of AI implementation.

*Currently, the system’s judgment of complex clinical situations is not sufficiently intelligent, sometimes resulting in false positives or false negatives.*

[N5]

*AI is always cold and impersonal; it will never replace us.*

[N1]

*Although protective measures have been implemented, the risk of information leakage persists.*

[N3]

Participants also reported that conflicts between AI-generated recommendations and nurses’ clinical judgment were difficult to avoid. They emphasized that clinical judgment should remain paramount and expressed concern that excessive reliance on AI-driven alert systems might weaken nurses’ fundamental skills and clinical reasoning abilities over time.

*Excessive reliance on smart alert systems may lead to the deterioration of nurses' fundamental skills and the weakening of clinical reasoning…*

[N3]

Cost was identified as another major barrier to AI implementation in ICUs. Participants consistently highlighted that adopting AI required substantial financial investment. These financial pressures were perceived as limiting the feasibility and long-term sustainability of AI implementation.

*Money is the biggest obstacle. It requires substantial capital investment, including hardware procurement, system development, and ongoing maintenance.*

[N5]

#### Facilitators: Relative Advantages and Design Quality and Packaging

Participants consistently perceived clear relative advantages associated with AI implementation in ICUs, which functioned as important facilitators. These advantages were reflected in multiple aspects of nursing practice, including improved nursing efficiency and convenience, mitigation of labor shortages, provision of information integration and decision support, improved nursing quality and patient safety, and optimization of teaching and training.

*There are advantages that can make our work more convenient.*

[N1]

*Third, it reduces labor costs by freeing nurses from repetitive, mechanical tasks.*

[N2]

*… AI will alert you to potential issues with the patient, prompting you to make decisions on care interventions…*

[N8]

*New graduate nurses can practice repeatedly in this risk-free environment (virtual simulation platform), enhancing both procedural proficiency and confidence in handling complex situations.*

[N3]

Regarding design quality and packaging, participants reported that some AI systems demonstrated favorable design features, such as the ability to integrate data from patient monitoring systems, ventilators, and infusion pumps into a unified analytical platform.

*Patient monitoring, ventilator data, and infusion pump information can be integrated together for analysis.*

[N10]

#### Neutral Influencing Factor: Trialability

Trialability was identified as a neutral influencing factor in the implementation of AI in ICUs. Participants reported that AI applications were commonly introduced through pilot or trial phases, allowing clinical teams to observe system performance in real-world settings and identify operational issues during early use.

*We encountered a new issue. When nurses were verifying information, their PDAs (Personal Digital Assistants) would ring, not only delaying tasks but also potentially causing errors. So, I made another change. I pushed the alert notifications to our watches.*

[N7]

*We're currently trialing the system, and we've noticed some lag. This is likely because our hospital has high staffing, with nurses stationed right at the bedside. So, our nurses often detect changes in a patient’s condition earlier than the AI does.*

[N9]

### Outer Setting

Within the outer setting domain of the CFIR, all 3 identified constructs (patient needs and resources, cosmopolitanism, and external policies and incentives) were identified as facilitators (Table 3).

Patients’ needs and resources emerged as important facilitators. Participants indicated that the rapidly changing and complex conditions of ICU patients placed high demands on nurses’ clinical knowledge and judgment. In addition to clinical demands, participants noted that patients’ emotional experiences and subjective feelings were not always adequately addressed in current practice.

*Because ICU patients' conditions change rapidly, it particularly challenges nurses' knowledge.*

[N5]

*We're all still in the exploratory stage, but I believe we need to pay more attention to the patients' feelings. While our current focus may be on research and development, we sometimes overlook how patients feel.*

[N8]

Cosmopolitanism was also identified as a significant facilitator. Most participants emphasized the importance of engaging with manufacturers and suppliers and strengthening collaboration with external organizations, such as technology companies and universities, to support AI development and implementation.

*The needs of manufacturers and suppliers are also crucial. We should collaborate with them on development and proactively communicate our requirements.*

[N1]

*Yes, this is a virtual simulation teaching system for suctioning procedures that we developed in collaboration with the university.*

[N3]

External policies and incentives further facilitated the implementation of AI. Participants emphasized that national-level policy guidance, together with strong leadership attention, provided the confidence to adopt AI technologies in ICUs.

*National policy guidance and a high level of leadership attention are crucial, as they give us confidence.*

[N4]

### Inner Setting

Within the inner setting domain, 4 constructs were identified, including 2 barriers and 2 facilitators influencing AI implementation in ICUs (Table 3).

#### Barriers: Structural Characteristics and Networks and Communications

Structural characteristics were identified as barriers to AI implementation. Participants reported a shortage of multidisciplinary professionals with integrated expertise in health care, nursing, and IT, which limited organizational capacity to support AI-related initiatives. Although IT teams existed at both the departmental and hospital levels, the lack of professionals capable of bridging clinical and technical domains constrained effective collaboration and implementation.

*To understand health care, nursing, and information technology—talent in this area is in short supply… I think it would be ideal if there were people who understand both healthcare and information technology…*

[N7]

Networks and communication were also perceived as barriers. Participants described challenges in interdisciplinary communication, particularly between clinical staff and IT professionals, which hindered effective collaboration.

*Communication among their peers might be more effective. Some individuals won't approach you (the head nurse) directly; they'd rather discuss things with their peers, sharing what’s not quite right…*

[N8]

*When I communicate with someone who only understands information, we encounter communication barriers. The things they produce end up with many bugs, requiring constant fixing and reworking…*

[N7]

#### Facilitators: Implementation Climate and Readiness for Implementation

Implementation climate was identified as a facilitator of AI implementation in ICUs. Participants emphasized that performance-linked incentives, such as bonuses, enhanced nurses’ motivation and AI literacy by recognizing and rewarding their engagement in AI-related initiatives. Additionally, role modeling and team-based learning were perceived to foster a supportive environment in which early adopters gained hands-on experience and subsequently promoted AI use among their colleagues.

*Particularly through bonuses, this will drive the enhancement of nurses' AI literacy.*

[N2]

*Gather nurses who are interested and have ideas, allowing them to learn and apply firsthand, gain experience, and then lead their colleagues to foster a positive atmosphere of learning and application.*

[N4]

Readiness for implementation was also identified as a facilitator. Participants emphasized that strengthening AI-related education and training enhanced nurses’ preparedness to engage with AI systems. Leadership engagement was viewed as particularly critical, as leaders’ visions and attitudes toward AI directly influenced organizational support and prioritization of AI implementation.

*Strengthen nurse training, with a greater emphasis on artificial intelligence.*

[N4]

*The vision and perspective of leaders influence the adoption of artificial intelligence.*

[N2]

### Characteristics of Individuals

The characteristics of individuals domain focused on personal attributes of nurses that influence AI implementation. Three constructs were identified as facilitators, including self-efficacy, individual stage of change, and other personal attributes (Table 3).

Self-efficacy was identified as a facilitator. Participants reported that nurses generally showed a positive attitude toward AI and actively sought opportunities to use it, reflecting high confidence in engaging with new tools.

*Nurses seem quite receptive to artificial intelligence, with many actively seeking to use it, so the acceptance rate is fairly good.*

[N2]

Individual stage of change was also identified as a facilitator. Participants indicated that the broader organizational and societal momentum toward AI adoption created shared expectations among nurses, encouraging proactive engagement and uptake of AI practices.

*When everyone around you is doing something, and you're the only one who doesn't think that way, it might just feel like you're the odd one out…*

[N7]

Other personal attributes further facilitated the implementation of AI. Participants reported that younger nurses were particularly quick to adapt and proactive in using AI technologies, with some even pursuing additional education to strengthen their expertise in AI.

*One nurse even considered quitting to pursue a master’s degree in artificial intelligence.*

[N9]

*Young nurses, in particular, adapt quickly to new technologies and are eager to learn and use them proactively.*

[N3]

### Process

The process domain encompassed activities and strategies related to AI implementation in ICUs. Four constructs were identified, including 3 facilitators and 1 neutral influencing factor (Table 3).

#### Facilitators: Planning, Engaging, and Executing

Planning was identified as a facilitator of AI implementation. Nurse managers anticipated that AI could optimize clinical processes such as delirium prevention, early mobilization, pressure injury detection, and quality control. Participants also highlighted the potential of logistics robots, intelligent scheduling systems, and medical large language models to support routine care, decision-making, and nursing education.

*I can think of things like delirium prevention and intervention, remote visits, assisting patients with early mobilization, pressure ulcer identification, and also quality control support.*

[N1]

*We look forward to developing a professional medical large language model that can help nurses quickly retrieve the latest clinical practice guidelines, automatically generate personalized care plans, and even provide decision support in emergency situations.*

[N3]

*Could robots replace family members or medical staff in keeping conscious patients’ company or chatting with them? There are actually some thoughts on this…*

[N7]

*We could use AI to develop case studies or conduct standardized training and exercises.*

[N11]

Participants further suggested that integrating the use of AI into departmental requirements and mandating its application in specific scenarios could promote adoption. Sending key nurses to observe and learn from proven practices in advanced units was also perceived as beneficial in strengthening implementation effectiveness and sustaining engagement.

*The focus now is more on how to implement artificial intelligence, which may require identifying specific areas where AI is necessary and mandating its use.*

[N2]

*Key personnel can be dispatched to visit and learn from the proven practices of advanced units.*

[N4]

Executing was also identified as a facilitator. Participants emphasized implementation of clear communication with patients and families regarding potential risks and establishing strict data management mechanisms to ensure that only authorized medical staff could access patient information.

*We will clearly inform patients and their families of potential risks and have them sign a specific informed consent form. At the same time, we have established a strict data management mechanism to ensure that only relevant medical staff can access the corresponding patient data.*

[N3]

#### Neutral Influencing Factor: Reflecting and Evaluating

Reflecting and evaluating were identified as neutral influencing factors in the implementation of AI in ICUs. Participants reported that although efforts to assess and review AI-related practices were underway, these processes remained exploratory and were not yet fully effective.

*We're currently exploring this area, but I don't think we're doing a particularly good job yet.*

[N7]

## Discussion

### Principal Findings

Guided by the CFIR framework, this study explored the barriers and facilitators to implementing AI in ICUs from the perspective of nurse managers across 7 geographic regions in China. A total of 5 barriers, 13 facilitators, and 2 neutral influencing factors were identified across all 5 CFIR domains. Overall, facilitators were reported more frequently than barriers, suggesting that nurse managers generally perceived substantial potential for AI to support ICU nursing practice in China. Beyond identifying barriers and facilitators within the CFIR domains, the study findings suggest that there may be a gap between the positive attitudes toward the implementation of AI in ICUs and the practical readiness. Many of these determinants appeared to reflect contextual characteristics specific to the Chinese health care environment, including strong policy support for digital health, hierarchical leadership structures, and the central role of nurse managers in coordinating interdisciplinary collaboration and guiding frontline adoption.

Within the intervention characteristics domain, relative advantage and design quality and packaging emerged as the most prominent facilitators of AI implementation. These findings were consistent with previous studies in general nursing and clinical settings, which have similarly reported perceived benefits of AI in improving efficiency, clinical decision support, and workflow optimization [13,30-32]. Similar positive perceptions have also been reported in studies on AI-enabled wearable technologies [33] and AI−internet of things health care services [16]. However, our findings further indicated that perceived usefulness alone was insufficient for ICU implementation. This finding suggests that CFIR intervention characteristics may require contextual interpretation in AI-enabled critical care, where perceived advantage is closely linked not only to efficiency but also to algorithmic reliability, patient safety, clinical accountability, and continuous system evolution. Compared with non-ICU settings, uncertainty regarding algorithmic reliability may be perceived more seriously in ICUs because even small errors or delays may affect patient safety [34,35]. In ICUs, AI systems must be reliable, interpretable, adaptable, and compatible with fast-paced clinical workflows. Participants were particularly concerned that algorithmic errors, delayed alerts, or poor contextual adaptation could directly affect patient safety. This concern may be more pronounced in ICUs than in general wards because clinical deterioration can occur rapidly and decisions are often time-sensitive [36,37].

Ethical and humanistic concerns emerged as important issues in this study. Previous studies have highlighted privacy risks, ethical uncertainty, overreliance on AI, and threats to professional autonomy as barriers to implementation [30,38,39]. In this study, nurse managers raised similar concerns. Participants emphasized that AI should not replace nurses’ clinical judgment or weaken humanistic care. Instead, AI should support nurses in making timely and informed decisions while preserving patient-centered care [40]. This finding highlights a tension that may be particularly salient in ICU settings between technological efficiency and ethical-humanistic responsibilities. Meanwhile, the boundaries of human-machine collaboration need to be clearly defined before AI can be safely embedded into ICU workflows.

Cosmopolitanism and external policies and incentives emerged as facilitators. Participants emphasized the value of collaboration with technology companies, universities, and interdisciplinary teams. This finding is consistent with international literature, indicating that successful AI implementation in health care depends on cooperation among clinical, technical, managerial, and regulatory stakeholders [41,42]. In China, strong governmental support for digital health and AI innovation provides legitimacy and momentum for AI adoption in ICUs. Such policy support may accelerate early-stage implementation by mobilizing resources and encouraging institutional engagement. However, the transferability of these findings to other health care systems should be interpreted cautiously. China’s health care system is characterized by strong policy guidance and relatively hierarchical organizational structures, which may facilitate top-down implementation.

Nurse managers emphasized that leadership vision strongly influenced the prioritization of AI implementation and resource allocation. These findings align with evidence from non-ICU settings, where leadership and organizational culture are consistently identified as critical determinants of innovation uptake [13,38]. Our findings add to this perspective by suggesting that nurse managers serve not only as organizational leaders but also as boundary-spanning actors who connect multiple implementation domains. ICU work is characterized by high workload intensity, rapid decision-making, and close interprofessional dependency. Nurse managers function as boundary spanners who connect multiple implementation domains, translating organizational priorities into clinical practice through coordination, resource allocation, and capability building. In this sense, AI implementation in ICUs is not only a technical process but also an organizational change process.

Training and education were also identified as essential facilitators. Consistent with prior studies, insufficient AI literacy [43] and limited digital competence [44] can hinder adoption among nurses and nursing students. Targeted training and continuing education programs can effectively promote the implementation of AI in nursing practice [45,46]. Notably, participants proposed incorporating AI applications into departmental requirements and mandating their use in specific scenarios to promote implementation. However, mandatory implementation strategies should be applied cautiously, as premature or overly rigid requirements may provoke resistance if AI systems are not yet sufficiently mature, interpretable, or trusted by frontline nurses. Generational differences were also observed. Younger nurses appeared more adaptable to AI, whereas senior nurses tended to be more cautious. This suggests the need for tailored training strategies and intergenerational learning models [13], with younger nurses potentially serving as AI champions. At the same time, the lack of nursing informatics professionals was identified as a barrier. Currently, few regions have incorporated nursing informatics into the required curriculum for the foundational nursing stage [40]. Nursing administrators should therefore advocate for the systematic integration of informatics and AI-related competencies into nursing education and continuing professional development.

Overall, while many facilitators and barriers are shared with general health care settings, AI implementation in ICUs is not simply an extension of general nursing AI adoption. ICU environments amplify concerns about safety, reliability, time sensitivity, ethical accountability, and workflow compatibility. These findings may inform the contextual application of the CFIR framework by illustrating how implementation determinants can be interpreted in high-risk clinical environments such as ICUs. Specifically, this study suggests a potential gap between positive implementation attitudes and actual organizational preparedness for AI adoption. Although supportive policies, leadership engagement, positive implementation climate, and professional self-efficacy were identified as important facilitators, challenges related to technological adaptation, resources, governance, and interdisciplinary collaboration may limit practical implementation. At the same time, issues such as algorithmic reliability, accountability mechanisms, transparency, and human-centered care may constitute cross-dimensional factors that affect multiple areas of implementation. Traditional classification frameworks may not be fully sufficient to provide a comprehensive explanation. Further research is needed to clarify how AI-specific dimensions can be integrated into implementation frameworks. Interestingly, this study identified more facilitators than barriers. This finding should be interpreted in light of the participants’ managerial roles. These participants may be more familiar with organizational strategy and innovation initiatives and therefore hold a more positive attitude toward the application of AI. Their managerial position provided unique insights into organizational readiness, leadership engagement, and contextual conditions that are often less visible from frontline perspectives. Consequently, while frontline nurses may place greater emphasis on usability, workload, and workflow disruption, nurse managers highlighted broader organizational and implementation issues that are essential for successful AI integration.

### Implications for Practice and Research

The findings of this study have important implications for ICU nursing management, policymakers, and technology developers. For nursing leaders, strengthening AI-related education and cultivating interdisciplinary talent are essential. Nursing leaders should develop interdisciplinary collaboration mechanisms and ensure that clinical requirements are effectively communicated to technical teams. Incentive mechanisms and role modeling strategies can further foster a supportive climate. Nursing organizations should also develop clear guidelines for the safe and appropriate use of AI technologies in clinical practice. For policymakers, supportive regulatory frameworks, targeted funding incentives, and standardized guidelines for AI governance and data security are critical to addressing cost barriers and privacy concerns. At the developer level, improving algorithm adaptability, interpretability, usability, and clinical integration may be critical to enhancing trust and sustainable adoption.

Future research should incorporate broader stakeholder perspectives, including frontline nurses, physicians, IT professionals, and patients, and should combine qualitative insights with quantitative evaluations of implementation outcomes. Longitudinal studies and mixed methods approaches will also be valuable for examining how AI implementation experiences change as technologies mature and are integrated into routine clinical practice. Theoretical research and empirical validation are also needed to clarify how these AI-specific dimensions can be integrated into implementation frameworks. Intervention studies testing context-sensitive implementation strategies will also be valuable in advancing evidence-based integration of AI in critical care settings.

### Limitations

Several limitations should be noted. First, the sample size was relatively small, and all participants were from China. Although we included participants across 7 geographic regions to capture variation in implementation contexts, selection bias may still exist. As health care systems, organizational structures, and implementation environments vary across countries, the transferability of these findings to other settings requires further investigation through studies conducted in diverse health care contexts. Second, this study focused specifically on the perspectives of nurse managers and did not include other key stakeholders, such as frontline nurses. As key decision-makers and coordinators within ICU nursing systems, nurse managers play an important role in shaping implementation priorities, facilitating interdisciplinary collaboration, allocating resources, and supporting organizational change. Their perspectives are therefore particularly valuable for understanding organizational and contextual determinants influencing AI implementation, which was the primary focus of this study. However, the role and position of nurse managers may introduce a hierarchical or managerial bias. Future research should incorporate multiple stakeholder groups, particularly frontline nurses, to obtain a more comprehensive understanding of the implementation of AI determinants in ICU settings. Third, AI implementation remains at an early stage, and perceptions may evolve as technologies mature. Finally, CFIR-guided inquiry and cross-language translation may have influenced theme emergence and nuanced meaning despite efforts to ensure analytic rigor.

### Conclusions

This study explored facilitators and barriers to AI implementation in ICUs from the perspective of nurse managers. The findings suggest that AI implementation is influenced by multiple determinants across technical, organizational, workforce, and process domains, as reflected in the CFIR framework. In particular, intervention characteristics and inner setting factors appear especially salient in ICUs. Nurse managers held a cautiously optimistic attitude toward AI implementation in ICU settings. They recognized its potential to improve clinical efficiency, decision support, workflow optimization, and patient safety. However, they also expressed concerns regarding limited humanistic care, ethical risks, and insufficient regulatory oversight. These challenges highlight the need for stronger governance systems, structured interdisciplinary collaboration, and targeted training for ICU nursing staff to support safe and sustainable AI integration in critical care.

## Supplementary material

10.2196/96172 Multimedia Appendix 1 Semistructured interview guide based on the Consolidated Framework for Implementation Research.

10.2196/96172 Multimedia Appendix 2 A summary of the barriers and facilitators of AI implementation grouped by Consolidated Framework for Implementation Research.

10.2196/96172 Checklist 1 COREQ checklist.

## Abbreviations

CFIR: Consolidated Framework for Implementation Research
COREQ: Consolidated Criteria for Reporting Qualitative Research
ICU: intensive care unit
