# Supplementary material for: Barriers and Facilitators to AI Implementation in Intensive Care Units in China: Qualitative Study Among Nurse Managers
Source: J Med Internet Res. 2026 Aug 18;28:e96172. doi: 10.2196/96172 (PMC13484927; doi:10.2196/96172)
Supplement: Supplementary file 1 [file jmir-v28-e96172-s001.docx]

**Supplementary Material 1 Semi-Structured Interview Guide Based on the CFIR Framework**

**I. Intervention Characteristics**

From your perspective, what types of artificial intelligence (AI) applications are currently used or being explored in your ICU?

How would you describe the relative advantages of AI compared with traditional nursing practices?

Do you trust the recommendations or alerts provided by current AI systems? How would you rate their accuracy and reliability? Have you ever encountered situations where you felt the AI's judgment was unreasonable?

To what extent do you think current AI systems are adaptable to different clinical scenarios or hospital contexts?

Have you encountered challenges related to system customization or contextual fit?

How do you perceive the complexity of AI systems in daily nursing practice? When using AI systems, do you feel they increase or decrease your cognitive load? For example, do you need more time to verify the AI's results, or do they actually save you time? To what extent do they disrupt your existing workflow?

Are there difficulties in understanding, operating, or interpreting AI outputs?

What are your views on the costs associated with implementing AI in the ICU (e.g., financial, time, training burden)?

How would you evaluate the design quality and usability of existing AI systems?

Has your ICU conducted pilot testing or trial implementation of AI systems? What impact did this have on your nursing management model and strategies?

What did you learn from these trial experiences?

What ethical, privacy, or patient safety concerns do you think may arise during AI implementation? How are these risks managed?

**II. Outer Setting**

In your opinion, how do ICU patients’ needs influence the adoption of AI technologies?

Do AI systems help address rapid condition changes or patient-centered care needs?

How does collaboration with external organizations (e.g., technology companies, universities, manufacturers) affect AI implementation in your ICU?

How do national or regional policies, regulations, or incentives influence decisions about adopting AI in your hospital?

**III. Inner Setting**

How would you describe your ICU’s organizational structure in supporting AI implementation?

How effective are communication and collaboration between nurses and technical teams during AI implementation?

What is the overall implementation climate in your ICU regarding AI adoption?

Are there incentives or expectations that encourage AI use?

How prepared do you think your ICU is for implementing AI?

What resources, training, or leadership support are available?

**IV. Characteristics of Individuals**

How do nurses in your ICU generally perceive AI technologies?

How confident are nurses in using AI systems in clinical practice?

What factors influence their confidence?

Have you observed differences among nurses (e.g., age, experience, educational background) in accepting or adopting AI?

**V. Process**

How was AI implementation planned in your ICU?

Who were the key individuals involved in promoting AI adoption?

How were nurses engaged during the process?

What functions or improvements would you like to see in future AI systems in ICU nursing?
