# Supplementary material for: Barriers and Facilitators to AI Implementation in Intensive Care Units in China: Qualitative Study Among Nurse Managers
Source: J Med Internet Res. 2026 Aug 18;28:e96172. doi: 10.2196/96172 (PMC13484927; doi:10.2196/96172)
Supplement: Supplementary file 2 [file jmir-v28-e96172-s002.docx]

**Supplementary Material 2 A summary of the barriers and facilitators of AI implementation grouped by CFIR.**

| **CFIR domain** | **CFIR constructs** | **Items** | **Partial illustrative quotes** |
| --- | --- | --- | --- |
| Intervention characteristics | Relative advantage (+) | Enhancing nursing efficiency and convenience | There are advantages that can make our work more convenient. (N1) |
|  |  |  | (AI) possesses powerful and stable capabilities, capable of simultaneously processing massive amounts of data without being affected by fatigue or emotions. (N2) |
|  |  |  | The most noticeable improvement is that the entire workflow has been optimized. (N3) |
|  |  | Mitigating labor shortages | Third, it reduces labor costs by freeing nurses from repetitive, mechanical tasks. (N2) |
|  |  |  | The current bed-to-nurse ratio makes it difficult to guarantee one-on-one care, but AI can compensate for the shortage of human resources. (N5) |
|  |  | Providing information integration and decision support | The patient's vital signs, laboratory results, and imaging data are all shared in real time via the network, allowing specialists to access them at any time. This significantly enhances the efficiency and accuracy of nursing consultations. (N3) |
|  |  |  | Through AI, he can determine the risk of sepsis in this patient, such as when critical values are detected... AI will alert you to potential issues with the patient, prompting you to make decisions on care interventions... (N8) |
|  |  | Enhancing nursing quality and patient safety | The nursing team leader wears a watch. If an alarm goes off and isn't responded to promptly, the leader knows the assigned nurse is occupied and unable to attend immediately. The leader can then step in to handle the situation. (N5) |
|  |  |  | After the doctor prescribes medication, the smart medicine cabinet automatically dispenses the drugs, eliminating the need for daily inventory checks. (N8) |
|  |  |  | We started this during the COVID-19 pandemic. When entering the isolation area via video monitoring... you must pass through that (camera-equipped) zone to capture various images. It checks whether you're wearing your mask and cap properly, and whether your hand hygiene meets our requirements. (N10) |
|  |  | Optimizing teaching and training | New graduate nurses can practice repeatedly in this risk-free environment (virtual simulation platform), enhancing both procedural proficiency and confidence in handling complex situations. (N3) |
|  |  |  | In our nursing practice, we primarily utilize artificial intelligence technology to generate teaching scenario designs and virtual case studies, while also serving as a learning tool for graduate nurses. (N4) |
|  | Adaptability (-) | System optimization and algorithm iteration | (False alarms) They will occur initially, but as the volume of data increases, the frequency of false alarms will decrease. (N1) |
|  |  |  | If conflicts frequently occur in certain scenarios, it indicates potential shortcomings in the algorithm. We should document these instances and provide feedback to the technical team for optimization. (N2) |
|  |  | Scenario adaptation and adjustment | Software that works well at one hospital may not necessarily be suitable for your own hospital. (N8) |
|  | Trialability (±) | | We encountered a new issue. When nurses were verifying information, their PDAs (Personal Digital Assistants) would ring, not only delaying tasks but also potentially causing errors. So I made another change. I pushed the alert notifications to our watches. (N7). |
|  |  |  | We're currently trialing the system, and we've noticed some lag. This is likely because our hospital has high staffing, with nurses stationed right at the bedside. So our nurses often detect changes in a patient's condition earlier than the AI does. (N9) |
|  | Complexity (-) | Insufficient system algorithm and clinical adaptability | (laugh) Currently, the system's judgment of complex clinical situations is not sufficiently intelligent, sometimes resulting in false positives or false negatives. (N5) |
|  |  | Judgment conflicts and dependency risks | (Decision conflict) is a very real issue and indeed something that is difficult to avoid...The clinical nurse's judgment remains paramount. (N2) |
|  |  |  | Excessive reliance on smart alert systems may lead to the deterioration of nurses' fundamental skills and the weakening of clinical reasoning. (N3) |
|  |  |  | No matter how much it (AI) thinks, it can never replace your own thinking...(N11) |
|  |  | Humanistic concern and ethical challenges | Artificial intelligence is always cold and impersonal; it will never replace us. (N1) |
|  |  |  | This raises issues of data security and patient privacy management. Ensuring the security of medical data poses a significant challenge. (N2) |
|  |  | Data security and privacy protection | All intelligent systems operate online, involving vast amounts of patient privacy data. Although protective measures have been implemented, the risk of information leakage persists. (N3) |
|  |  | System integration and data organization challenges | But that's a whole new problem. No one is here to help us organize this data. (N8) |
|  | Design quality and packaging (+) | | Patient monitoring, ventilator data, and infusion pump information can be integrated together for analysis. (N10) |
|  | Cost (-) | | (laugh) Money is the biggest obstacle! It requires substantial capital investment, including hardware procurement, system development, and ongoing maintenance. (N5) |
| Outer setting | Patient needs and resources (+) | | Because ICU patients' conditions change rapidly, it particularly challenges nurses' knowledge. (N5) |
|  |  |  | We're all still in the exploratory stage, but I believe we need to pay more attention to the patients' feelings. While our current focus may be on research and development, we sometimes overlook how patients feel. (N8) |
|  | Cosmopolitanism (+) | | The needs of manufacturers and suppliers are also crucial. We should collaborate with them on development and proactively communicate our requirements. (N1) |
|  |  |  | Yes, this is a virtual simulation teaching system for suctioning procedures that we developed in collaboration with the university. (N3) |
|  | External policies and incentives (+) | | National policy guidance and a high level of leadership attention are crucial, as they give us confidence. (N4) |
| Inner setting | Structural characteristics (-) | Shortage of multidisciplinary nursing information professionals | To understand healthcare, nursing, and information technology—talent in this area is in short supply... I think it would be ideal if there were people who understand both healthcare and information technology...(N7) |
|  |  | Information technology team development | Our department, along with the hospital, has an IT team...(N7) |
|  | Networks and communications (-) | Interdisciplinary collaboration | These engineers are detached from clinical practice, so he now frequently attends our meetings to hear our ideas and draw inspiration from us. (N9) |
|  |  | Informal communication | Communication among their peers might be more effective. Some individuals won't approach you (the head nurse) directly; they'd rather discuss things with their peers, sharing what's not quite right... (N8) |
|  |  | Interdisciplinary communication barriers | When I communicate with someone who only understands information, we encounter communication barriers. The things they produce end up with many bugs, requiring constant fixing and reworking... (N7) |
|  | Implementation climate (+) | Incentive bonuses and system orientation | Particularly through bonuses, this will drive the enhancement of nurses' AI literacy. (N2) |
|  |  |  | Then these individuals (promoting AI implementation) are certainly different in this regard, and that should definitely be reflected in their bonuses. (N7) |
|  |  | Role model influence and team learning | Gather nurses who are interested and have ideas, allowing them to learn and apply firsthand, gain experience, and then lead their colleagues to foster a positive atmosphere of learning and application. (N4) |
|  | Readiness for implementation (+) | AI knowledge training | Strengthen nurse training, with a greater emphasis on artificial intelligence. (N4) |
|  |  | Leadership engagement | The vision and perspective of leaders influence the adoption of artificial intelligence. (N2) |
| Characteristics of individuals | Self-efficacy (+) | | Nurses seem quite receptive to artificial intelligence, with many actively seeking to use it, so the acceptance rate is fairly good. (N2) |
|  |  |  | The government has placed significant emphasis on this matter, so public confidence in it remains very strong. (N8) |
|  | Individual stage of change (+) | | When everyone around you is doing something, and you're the only one who doesn't think that way, it might just feel like you're the odd one out... (N7) |
|  | Other personal attributes (+) | | One nurse even considered quitting to pursue a master's degree in artificial intelligence. (N9) |
|  |  |  | Young nurses, in particular, adapt quickly to new technologies and are eager to learn and use them proactively. (N3) |
| Process | Planning (+) | Clinical nursing practice | I can think of things like delirium prevention and intervention, remote visits, assisting patients with early mobilization, pressure ulcer identification, and also quality control support. (N1) |
|  |  |  | That's too much. For instance, logistics robots that pick up and deliver items, assist with blood sugar control, and intelligently adjust pain relief and sedative medications. Then there's smart scheduling... If that were possible, it would really save us a lot of trouble (laughs). (N5) |
|  |  |  | We look forward to developing a professional medical large language model that can help nurses quickly retrieve the latest clinical practice guidelines, automatically generate personalized care plans, and even provide decision support in emergency situations. (N3) |
|  |  |  | We hope that in the future, more intelligent robots will assist with basic care tasks such as turning patients, cleaning, and feeding. This would free nurses from heavy physical labor, allowing them to focus more on specialized nursing care. (N3) |
|  |  | Nursing environment and humanistic optimization | Could robots replace family members or medical staff in keeping conscious patients company or chatting with them? There are actually some thoughts on this...(N7) |
|  |  | Education, training, and scientific research innovation | We could use AI to develop case studies or conduct standardized training and exercises. (N11) |
|  | Engaging (+) | | The focus now is more on how to implement artificial intelligence, which may require identifying specific areas where AI is necessary and mandating its use. (N2) |
|  |  |  | I believe this (use of AI) is somewhat related to the department's requirements. (N7) |
|  |  |  | Key personnel can be dispatched to visit and learn from the proven practices of advanced units. (N4) |
|  | Executing (+) | | You may have incorporated this process into your management duties, and now you may also need to oversee training on new technologies. (N6) |
|  |  |  | We will clearly inform patients and their families of potential risks and have them sign a specific informed consent form. At the same time, we have established a strict data management mechanism to ensure that only relevant medical staff can access the corresponding patient data. (N3) |
|  | Reflecting and evaluating (±) | | We're currently exploring this area, but I don't think we're doing a particularly good job yet. (N7) |
