# Supplementary material for: Barriers and Facilitators to AI Implementation in Intensive Care Units in China: Qualitative Study Among Nurse Managers
Source: J Med Internet Res. 2026 Aug 18;28:e96172. doi: 10.2196/96172 (PMC13484927; doi:10.2196/96172)
Supplement: Supplementary file 3 [file jmir-v28-e96172-s003.docx]

**Supplementary Material 2 COREQ (Consolidated criteria for Reporting Qualitative research) Checklist**

| **No.** | **Topic** | **Guide Questions/ Description** | **Reported on Page No.** |
| --- | --- | --- | --- |
| **Domain 1: Research team and reflexivity** | | | |
| *Personal characteristics* | | | |
| 1 | Interviewer/facilitator | Which author/s conducted the interview or focus group? | Page 5 |
| 2 | Credentials | What were the researcher’s credentials? | Master, BSc |
| 3 | Occupation | What was their occupation at the time of the study? | Page 5 |
| 4 | Gender | Was the researcher male or female? | Both |
| 5 | Experience and training | What experience or training did the researcher have? | Page 5 |
| *Relationship with participants* | | | |
| 6 | Relationship established | Was a relationship established prior to study commencement? | Page 5 |
| 7 | Participant knowledge of the interviewer | What did the participants know about the researcher? e.g. personal goals, reasons for doing the research | Page 4 |
| 8 | Interviewer characteristics | What characteristics were reported about the inter viewer/facilitator? e.g. Bias, assumptions, reasons and interests in the research topic | Page 6 |
| **Domain 2: Study design** | | | |
| *Theoretical framework* | | | |
| 9 | Methodological orientation and Theory | What methodological orientation was stated to underpin the study? e.g. grounded theory, discourse analysis, ethnography, phenomenology, content analysis | Page 6 |
| *Participant selection* | | | |
| 10 | Sampling | How were participants selected? e.g. purposive, convenience, consecutive, snowball | Page 4 |
| 11 | Method of approach | How were participants approached? e.g. face-to-face, telephone, mail, email | Page 4 |
| 12 | Sample size | How many participants were in the study? | Page 9 |
| 13 | Non-participation | How many people refused to participate or dropped out? Reasons? | Page 9 |
| *Setting* | | | |
| 14 | Setting of data collection | Where was the data collected? e.g. home, clinic, workplace | Page 5 |
| 15 | Presence of non participants | Was anyone else present besides the participants and researchers? | No |
| 16 | Description of sample | What are the important characteristics of the sample? e.g. demographic data, date | Page 9 |
| *Data collection* | | | |
| 17 | Interview guide | Were questions, prompts, guides provided by the authors? Was it pilot tested? | Page 5 |
| 18 | Repeat interviews | Were repeat inter views carried out? If yes, how many? | No |
| 19 | Audio/visual recording | Did the research use audio or visual recording to collect the data? | Page 5 |
| 20 | Field notes | Were field notes made during and/or after the inter view or focus group? | Page 5 |
| 21 | Duration | What was the duration of the inter views or focus group? | Page 9 |
| 22 | Data saturation | Was data saturation discussed? | Page 5 |
| 23 | Transcripts returned | Were transcripts returned to participants for comment and/or correction? | No |
| **Domain 3: analysis and findings** | | | |
| *Data analysis* | | | |
| 24 | Number of data coders | How many data coders coded the data? | Page 7 |
| 25 | Description of the coding tree | Did authors provide a description of the coding tree? | Page 6 |
| 26 | Derivation of themes | Were themes identified in advance or derived from the data? | Page 6 |
| 27 | Software | What software, if applicable, was used to manage the data? | Page 7 |
| 28 | Participant checking | Did participants provide feedback on the findings? | No |
| *Reporting* | | | |
| 29 | Quotations presented | Were participant quotations presented to illustrate the themes/findings? Was each quotation identified? e.g. participant number | Page 11-18 |
| 30 | Data and findings consistent | Was there consistency between the data presented and the findings? | Page 11-18 |
| 31 | Clarity of major themes | Were major themes clearly presented in the findings? | Page 11-18 |
| 32 | Clarity of minor themes | Is there a description of diverse cases or discussion of minor themes? | Page 11-18 |
